# Head or Figurehead?

**Published:** 1899-10

**Authors:** 


### HEAD OR FIGUREHEAD?

UNDER the above caption *Pediatrics*, of the issue of September 15th, advances an idea in connection with the christian science agitation which is worthy of serious consideration. The well-known fact that the christian science publishing firm, in Boston, has taken in over a million dollars for the seeress' books alone, tends to give color to *Pediatrics'* idea. The editorial is as follows :

It is conceivable that a devout christian woman, with a belief in the divine origin of her "mission," might found a religious sect; and that, in common with many other religious sects, seeming miracles of healing might be wrought through the faith of the believers. When, however, the visible head of a sect of this sort exhibits a financial ability suggestive of the manipulation of a syndicate of professional capitalists, the casual observer may well stand aghast at the prodigy of such a combination of saint and plutocrat. It is true that devoutness and dollar-getting are not incompatible, but adequate business training has always been a necessary requirement for the accumulation of millions; and in the absence of such a preparation the casual observer ought to be pardoned for imagining that the "head" of the new sect could be no more than a mere figurehead for a number of shrewd money-getting men. We do not, in this connection, refer to the fees charged by "healers." For if the ministrations of these functionaries are really a part of the offices of the church, it is as consistent to accept fees for healing as it would be in the case of weddings and other ceremonies, provided their operations are restricted to members of their own faith. We refer to the financial ability which can divert into coffers of the promoters a million (?) dollars obtained from the sale of a book published at nominal cost; and of a corresponding sum, perhaps, obtained from the sale of souvenir spoons; to say nothing of the thousands received annually from tuition fees, revenues from magazines, etc. Certain capitalists "like" medicine as an investment. Modern financiering is a game of grabbing the profits of others. Vast profits are derived from the sale of proprietary remedies, and from the legitimate trade in drugs. What a grand scheme to persuade people that all drugs are useless and injurious, and then to further persuade them to buy our books and mascot spoons, and to pay preposterous fees for a few weeks' instruction in science healing! Of late years the woman figurehead has done good service for medical capitalists. There is the mythical Mrs. Pinkham, for example; and there are a half dozen women figureheads for alleged cosmetic and hygienic remedies. Capitalists unblushingly hire a good-looking woman in her twenties to tell other women that she is forty years old and keeps young by using the many high-priced cosmetics which bear her name. Advertising does the rest. The ostensible head of the scientists similarly tells her following how to be healthy and wise. It is far from unlikely that a few years hence, when the christian science fad shall have been superseded by newer medical fads, the fact will come to light that this head of the scientists has been either the dupe or confederate of one or more shrewd capitalists.

---

THE memorial address upon the life and character of Lawson Tait, by Dr. Charles A. L. Reed, of Cincinnati, delivered at the recent meeting of the American Association of Obstetricians and Gynecologists, at Indianapolis, was an effort every way worthy of the occasion. Rarely has so appropriate a tribute been offered to the memory of a distinguished medical man, and the memorialist, who was at his best in voice and manner, did himself and his profession great credit in the address, which abounded in ornate and graceful rhetoric, in scholarly finish, and in historic accuracy. It is most interesting reading, but it should have been heard to be fully appreciated.

---

SUCH familiar and versatile literateurs as "Medico," "Æsculapius," "M.D.," etc., have started in to appoint the medical corps of the Pan-American Exposition through the medium of the daily newspaper correspondent's column.

---

JUDGING by the delay and inactivity of the Board of Aldermen in christian science matters, it would appear to the casual observer that some of the witch doctors had managed to land a "spell" in the ordinance committee room.

